# Development and Application of gSSRs Markers in *Macadamia* from Genomic Sequences

**DOI:** 10.3390/life16101618

**Published:** 2026-09-26

**Authors:** Can Cui, Guifang Yang, Yao Liu, Zijuan Yu, Guang Yang, Xiaoxian Ruan, Jinyun Zhang, Shijun Wu

**Affiliations:** 1 School of Horticulture and Food, Guangdong Eco-Engineering Polytechnic, Guangzhou 510520, China; cuicansurewin123@163.com (C.C.);; 2 School of Life Sciences, Sun Yat-sen University, Xingang Xi Lu 135, Guangzhou 510275, China

**Keywords:** *Macadamia*, gSSR markers, genetic diversity, population structure, cultivated germplasm

## Abstract

*Macadamia* (*Macadamia* spp.) is an economically important nut crop worldwide, but genomic simple sequence repeat (SSR) markers remain limited, especially for germplasm from South China, hindering comprehensive genetic diversity assessment and population structure analysis. In this study, we developed and characterized gSSR markers based on leaf genomic sequence data. A total of 76 primer pairs were initially designed, of which 65 were successfully amplified, and 18 highly polymorphic markers were selected for further analysis. Using these 18 gSSR markers, we genotyped 104 *Macadamia* individuals from 15 populations in South China. The markers detected a total of 118 alleles, with the number of alleles per locus ranging from 2.056 to 7.167 (mean = 2.911). The observed heterozygosity (H_o_) and expected heterozygosity (H_e_) varied from 0.556 to 0.697 and from 0.389 to 0.669, respectively. Analysis of molecular variance (AMOVA) revealed significant genetic differentiation among populations (*F*_ST_ = 0.304, *p* < 0.01), with 16.39% of the variation occurring between populations and 83.61% within populations. Population structure analysis based on Structure, PCoA, and UPGMA clustering consistently divided the 15 populations into two distinct genetic groups. Relative migration analysis revealed substantial gene flow among the four populations examined in this study, with bidirectional relative migration rates all exceeding 0.7. One outlier locus (MacChr13-2) showing a positive selection signal was identified, suggesting potential adaptive divergence. These newly developed gSSR markers provide a valuable tool for genetic diversity assessment, germplasm management and cultivar improvement, and marker-assisted breeding of *Macadamia*.

## 1. Introduction

Genetic diversity is fundamental to crop improvement, germplasm management, and the long-term adaptive capacity of cultivated plant populations [1,2]. Assessing the level and distribution of genetic variation within and among populations provides essential insights into demographic history, gene flow, genetic drift, and the effects of artificial selection [3]. Among the array of DNA-based marker systems available for such assessments, simple sequence repeats (SSRs) remain one of the most widely used tools, particularly for species lacking high-throughput genotyping platforms, owing to their high genomic abundance, codominant inheritance, multi-allelic nature, and good reproducibility [4]. SSRs are classified into genomic SSRs (gSSRs), derived from non-coding genomic regions, and expressed sequence tag SSRs (EST-SSRs), obtained from transcribed regions [5]. gSSRs generally display higher polymorphism and are therefore more informative for discriminating closely related cultivars and resolving fine-scale population structure [6]. The increasing availability of next-generation sequencing data and dedicated bioinformatics pipelines has greatly facilitated the cost-effective mining and development of gSSR markers from genomic sequences across a wide range of plant species [4,7].

*Macadamia* (*Macadamia* spp.), a member of the family Proteaceae, is native to the subtropical rainforests of southeastern Queensland and northeastern New South Wales, Australia [8]. The genus comprises four specie: *M. integrifolia*, *M. tetraphylla*, *M. ternifolia*, and *M. jansenii*, all of which are listed as vulnerable or endangered in their natural habitats owing to habitat loss and fragmentation. Commercial *Macadamia* production is based primarily on *M. integrifolia*, *M. tetraphylla*, and their interspecific hybrids, which are now cultivated in tropical and subtropical regions worldwide, including Australia, Hawaii, South Africa, Brazil, and China [9]. Since its domestication in the mid-nineteenth century, the global macadamia industry has relied on a relatively narrow genetic base, with most commercial cultivars tracing back to a small number of wild ancestors from the northern range of *M. integrifolia* [10]. Chromosome-scale genome assemblies for *M. integrifolia* and *M. tetraphylla* have recently become available, enhancing the genomic resources for marker development and genetic improvement of the genus [10,11]. This narrow genetic foundation underscores the importance of characterizing cultivated germplasm to guide breeding and the sustainable management of genetic resources.

Over the past two decades, molecular markers have been applied to assess genetic diversity and relationships in *Macadamia* germplasm. Studies of wild Australian populations have revealed geographically structured genetic variation in both *M. integrifolia* and *M. tetraphylla*, with substantial differentiation among regional groups and evidence of crop-to-wild gene flow where orchards abut natural populations [12,13,14]. In cultivated germplasm, SNP and silicoDArT markers have been used to characterize population structure and linkage disequilibrium in an Australian breeding population [15], while SSR markers have been employed to assess accessions in South Africa and Brazil [12,16]. However, these studies have focused predominantly on diversity estimation and DNA fingerprinting within germplasm collections; none has systematically examined population genetic structure, relative migration patterns, or outlier loci among cultivated macadamia populations in South China, where the industry has expanded rapidly in recent years. Moreover, the number of publicly available, validated gSSR markers for macadamia remains limited relative to other major tree crops, constraining comprehensive population genetic analyses.

To address these gaps, the present study had three objectives: (1) to develop and characterize a set of novel gSSR markers from macadamia genomic sequence data; (2) to assess the genetic diversity, population structure, and relative migration patterns among 104 cultivated macadamia individuals representing 15 populations from a planting base in South China; and (3) to screen for outlier loci potentially under selection. We hypothesized that cultivated populations derived from different parental combinations would exhibit detectable genetic structure despite the overall narrow genetic base of domesticated macadamia, and that the newly developed gSSR markers would reveal sufficient polymorphism to resolve these patterns. The resulting marker set and population genetic data are intended to provide a practical tool for *Macadamia* germplasm management, cultivar identification, and future marker-assisted breeding efforts.

## 2. Materials and Methods

### 2.1. Sample Collection and DNA Extraction

Fresh young leaves from 104 *Macadamia* (*Macadamia* spp.) individuals were collected from the planting base of Guangdong Eco-Engineering Polytechnic and immediately preserved in liquid nitrogen. Population sources with detailed parental information and their corresponding abbreviations are presented in Supplementary Table S1. Approximately 300 mg of fresh leaf tissue was weighed, combined with stainless steel grinding beads, frozen in liquid nitrogen, and homogenized using an MM400 automatic grinder (Retsch, Haan, Germany). Genomic DNA was extracted using a novel plant genomic DNA extraction kit (Aweidi Biotechnology Co., Ltd. Guangzhou, China). DNA concentration and purity were assessed using a NanoDrop 2000 spectrophotometer (Thermo Fisher Scientific, Waltham, MA, USA). DNA samples with concentrations > 100 ng/μL and purity (A260/280 > 2.00) were selected and stored at −20 °C for subsequent PCR amplification.

### 2.2. SSR Loci Identification and Primer Design

Genomic sequencing data used for SSR development were obtained from the NCBI Sequence Read Archive (SRA). The chromosome-scale genome assembly of *Macadamia* integrifolia cultivar HAES 741 (assembly SCU_Mint_v3) is available at NCBI under BioProject PRJNA593881, with GenBank assembly accession GCA_013358625.1 (RefSeq: GCF_013358625.1; WGS accession JAAEEG000000000). Raw genomic DNA reads are deposited in the NCBI Sequence Read Archive under accessions SRR10896963, SRR10896962, SRR10896961 (Illumina HiSeq 2500 paired-end), and SRR10896960 (PacBio RS II). Simple sequence repeats (SSRs) with di- and trinucleotide motifs were identified using MISA software (MISAv2.0), with thresholds of at least six repeats for dinucleotides and four repeats for trinucleotides. Primer pairs were designed using Premier v6.0 (Premier Biosoft International, Palo Alto, CA, USA) with the following criteria: primer length of 18–25 bp (optimal 20 bp), GC content of 40–60%, expected amplicon size of 100–400 bp, minimal formation of dimers and hairpin structures, and similar melting temperatures (Tm = 60 °C). A total of 76 primer pairs were designed, and detailed information is provided in Supplementary Table S2.

### 2.3. SSR Primer Screening, Amplification, and Validation

PCR conditions for SSR primers were optimized using genomic DNA from a single *Macadamia* individual in a 10 μL reaction system. The PCR amplification system was shown as Table 1.

The PCR program consisted of an initial denaturation at 94 °C for 4 min; followed by 33 cycles of denaturation at 94 °C for 30 s, annealing at 60 °C for 30 s, and extension at 72 °C for 30 s; with a final extension at 72 °C for 5 min. PCR products were evaluated by agarose gel electrophoresis, and primers yielding clear, single bands consistent with expected fragment sizes were selected. Finally, a total of 65 primer pairs were retained for subsequent polymorphism screening and genotyping.

### 2.4. gSSR Genotyping and Population Genetic Data Analysis

A total of 65 SSR primer pairs were initially used to assess polymorphism in eight randomly selected macadamia individuals. Based on polymorphism levels, 18 highly informative primer pairs were selected for genetic diversity analysis of all 104 individuals.

PCR amplification for SSR genotyping was conducted in a 10 μL reaction system containing 1 μL 10 × buffer (100 mM Tris-HCl, pH 9.0; 80 mM (NH_4_)2_S_O_4_; 100 mM KCl; 0.5% NP-40), 2.0 mM MgCl_2_, 25 μM of each dNTP, 0.5 μM forward and reverse primers, 0.5 U Taq DNA polymerase, 10 pmol fluorescently labeled dUTP (Fermentas, Burlington, ON, Canada), and approximately 20 ng template DNA. PCR amplification was performed using a DNA Engine thermal cycler (Bio-Rad, Hercules, CA, USA) under the following conditions: 94 °C for 4 min; 33 cycles of 94 °C for 30 s, 60 °C for 30 s, and 72 °C for 30 s; followed by a final extension at 72 °C for 5 min.

SSR fragments were detected using an ABI 3130xl Genetic Analyzer (Applied Biosystems, Carlsbad, CA, USA). For fragment analysis, 1 μL of PCR product was mixed with 9.34 μL Hi-Di formamide and 0.16 μL GeneScan 500 LIZ internal size standard (Applied Biosystems, CA, USA), denatured at 95 °C for 5 min, and immediately chilled on ice. Allele scoring was conducted using GeneMapper v4.0 software (Applied Biosystems, CA, USA) with a bin width of ±1 bp. Stutter artifacts (minor peaks 2–4 bp below the main peak, <15% of peak height) were identified and excluded from allele calling. All electropherograms were independently inspected by two researchers, and ambiguous or low-intensity samples were reamplified and regenotyped.

Null alleles were detected using Micro-Checker v2.2.3 based on 1000 Monte Carlo simulations. Population genetic parameters, including number of alleles (N_a_), observed heterozygosity (H_o_), expected heterozygosity (H_e_), polymorphism information content (PIC), and Shannon’s information index (I), were calculated using POPGENE v1.32. The number and percentage of polymorphic loci in each population were calculated using the Frequency module in GenAlEx v6.41. The number of private alleles was calculated using the R packages poppr 2.9.5, ade 4 1.7-22, and adegenet 2.1.10.

### 2.5. Genetic Differentiation and Population Structure Analysis Based on gSSRs

Analysis of molecular variance (AMOVA) was performed using Arlequin v3.5 to estimate genetic variation within and among macadamia populations. Population structure of 15 germplasm accessions was inferred using a Bayesian clustering approach implemented in STRUCTURE v2.3.4. The number of clusters (K) was set from 1 to 16, with 10 independent runs for each K value. Both the Markov Chain Monte Carlo (MCMC) iterations and burn-in period were set to 100,000.

The optimal K value was determined based on the ΔK method using STRUCTURE HARVESTER. CLUMPP and DISTRUCT were used to estimate average membership coefficients and visualize clustering results. Principal coordinate analysis (PCoA) was conducted using GenAlEx v6.5. An unweighted pair group method with arithmetic mean (UPGMA) phylogenetic tree based on Nei’s genetic distance was constructed using the R package gplots.

### 2.6. Relative Migration Rate Analysis Among Populations and Function Annotation

Relative migration rates among populations were estimated using the divMigrate function in the R package diveRsity (v1.9.90). The Nm estimator was used as an indicator of gene flow. Bootstrap analysis was conducted with 1000 replicates, and 95% confidence intervals were applied. A threshold of Nm = 0.1 was set for visualization, such that only gene flow values exceeding this threshold were displayed in the migration network. Pairwise relative migration rates among populations were thus obtained. Sequences contained SSR were compared against the KOG (Karyotic Orthologous Groups) database using BLASTX 2.12.0 with an E-value threshold of 10^−3^, and sequences were assigned to KOG functional categories based on their best hit.

## 3. Results

### 3.1. Genetic Diversity Analysis Based on gSSRs

The PCR amplification screening results for some individual *Macadamia* accessions are shown in Figure 1. The genetic diversity parameters of natural *macadamia* populations are presented in Table 2. A total of 118 alleles were detected across 18 primer pairs in macadamia individuals. The number of alleles (N_a_) ranged from 2.056 (CI) to 7.167 (CK), with a mean value of 2.911. The effective number of alleles (N_e_) was highest in population CK (4.141) and lowest in population CA (1.904), with an average of 2.320. Observed heterozygosity (H_o_) and expected heterozygosity (H_e_) ranged from 0.556 (CA) to 0.697 (CB) and from 0.389 (CA) to 0.669 (CK), respectively. Population CK exhibited the highest Shannon’s information index (I = 1.499), while population CA had the lowest (0.611). The allelic richness (A_r_) across populations varied from 0.305 (CI) to 2.803 (CX), averaging 1.975. The inbreeding coefficient (F_IS_) ranged from −0.455 (CJ) to 0.518 (XF), with a mean of 0.198. Except for populations CF and CJ, all other populations showed positive F_IS_ values. Private alleles (Np) were detected in seven populations, ranging from 0 (populations XF, CX, CB, CE, CF, CG, CH, and CI) to 53 (population CK). Analyses of N_e_, H_o_, H_e_, and I indicated that populations CA, CI, and CF had lower genetic diversity than the others. No evidence of null alleles was detected at any locus using Micro-Checker. Overall, all loci except Mac014 deviated significantly from Hardy–Weinberg equilibrium (HWE) (Table 3).

### 3.2. Base Composition of SSR Repeat Motifs in the Macadamia Genome

In this study, the SSR loci identified from the *Macadamia* genome comprised two types of repeat motifs. As shown in Figure 2a, both dinucleotide and trinucleotide repeats each contained only two types. Among the dinucleotide repeat motifs, CT/CT and GT/GT were the most abundant (26, 5.372%), while AT/AT was the least abundant (12, 2.479%). Among the trinucleotide repeat motifs, AAG/AAG was the most abundant (61, 15.496%), whereas CCT/CCT was the least abundant (12, 2.479%).

Functional prediction and classification of all SSR-containing chromosomal coding regions in the macadamia genome were performed using the KOG database. As shown in Figure 2b, a total of 286 SSRs were annotated into 24 KOG functional categories. Among these, amino acid transport and metabolism was the most abundant category, comprising 25 SSRs, accounting for 8.74% of all KOG-annotated SSRs. This was followed by lipid transport and metabolism, which contained 18 SSR loci, representing 6.29% of the total annotated SSRs. Post-translational modification, protein turnover, chaperones and signal transduction mechanisms contained 17 and 16 SSR loci, accounting for 5.94% and 5.59% of the total, respectively. In contrast, cell wall/membrane/envelope biogenesis was the least represented category, comprising only 1.05% of the total annotated SSRs. All identified SSR loci with their repeat motif type, repeat number, primer sequences and annealing temperature were represented in Supplementary Table S2.

### 3.3. Genetic Variation and Population Structure Analysis

Population structure was analyzed using the Bayesian approach implemented in STRUCTURE. As shown in Figure 3a, the results showed that ΔK exhibited pronounced changes at both K = 2 and K = 13, suggesting that K = 2 and K = 13 might be the optimal numbers of genetic clusters. However, at K = 13, the *Macadamia* populations were not effectively separated into distinct clusters. When K = 2, the 15 populations were clearly divided into two genetic groups, as shown in Figure 3b. Population CA was separated from the other populations at K = 2, suggesting that it carries a distinct genetic composition likely reflecting its unique parental background (male parent 900, female parent 695). However, this does not necessarily indicate an independent evolutionary lineage, and further sampling and marker density would be needed to resolve its status.

Principal coordinate analysis showed that the first two PCoA axes explained 11.74% and 6.18% of the total variation, respectively, as shown in Figure 4a. Furthermore, the UPGMA clustering analysis further validated the results obtained from STRUCTURE and PCoA, as shown in Figure 4b. Analysis of molecular variance (AMOVA) revealed significant genetic variation in *Macadamia* (*F*_ST_ = 0.304, *p* < 0.01), with genetic variation among and within populations accounting for 16.39% and 83.61% of the total variation, respectively (*p* < 0.01). In addition, AMOVA based on the two genetic clusters identified by STRUCTURE analysis showed that the degrees of freedom among and within populations were 14 and 89, respectively, with the corresponding variation accounting for 16.39% and 83.61% of the total variation, indicating lower genetic variation among populations relative to within populations (Table 4).

Analysis of relative migration rates based on Nm results (Figure 3) showed that among the 15 populations, pairwise bidirectional relative migration rates were relatively high for CK and CX (m_R_ > 0.7), CK and CH (m_R_ > 0.8), CX and CH (m_R_ > 0.8), and CM and CX (m_R_ > 0.7), indicating substantial gene flow among populations CK, CX, CH, and CM. Additionally, a certain degree of gene exchange was detected between CB and XF (m_R_ > 0.7). No significant gene exchange was detected among the other populations, namely CA, CE, SX, CF, CJ, CC, CD, CI, and CG. The specific values of pairwise relative migration rates among populations are provided in Table 5.

### 3.4. Identification of Outlier Loci

To further verify the reliability and accuracy of the developed primers, 18 SSR primer pairs with clear bands, high polymorphism, and good stability were selected for capillary electrophoresis and genotyping analysis of 104 *Macadamia* individuals. The 18 selected highly polymorphic SSR loci were functionally annotated against the KOG database and were mainly classified into Post-translational modification, protein turnover, chaperones, Translation, ribosomal structure and biogenesis, and Signal transduction mechanisms, indicating that these microsatellite containing loci participate in diverse biological processes underlying *Macadamia* growth and development (as shown in Supplementary Table S3).

In the BayeScan analysis, three loci were identified as balancing selection loci using the Bayesian approach, and one locus was identified as a positive selection locus (Figure 5a). The HIM test in Arlequin detected only one outlier locus with a significant *p*-value (Figure 5b), namely MacChr 13-2, which exhibited a potential positive selection signal.

## 4. Discussion

Expected heterozygosity (H_e_) is a primary measure of genetic diversity and is widely used to assess the diversity level of species [17]. In the present study, the mean H_e_ across all individuals was 0.4814, which falls within the H_e_ range (0.42–0.55) reported for introduced cultivated macadamia populations in major domestic production areas such as Yunnan and Guizhou, China, and is nearly identical to the H_e_ (0.492) reported by Zhang et al. for 42 *Macadamia* germplasm accessions from Yunnan [18]. This H_e_ value is markedly lower than those observed in wild Australian populations of *M. tetraphylla* and locally cultivated populations of *M. integrifolia* in China (H_e_ = 0.55–0.69) [12,19], and is also lower than the average genetic diversity (H_e_ = 0.626) of global commercial cultivars [20]. Across seed plants, the magnitude and partitioning of genetic diversity are strongly governed by life-history and breeding system traits: long lived, outcrossing, late-successional taxa generally retain most of their variability within populations, whereas annual, selfing and early successional taxa allocate a larger fraction of their variation among populations [21]. In *Macadamia*, the realized diversity is further shaped by intrinsic characteristics and external factors such as the reproductive system, genetic drift, introduction bottlenecks and environmental selection [21,22,23].

*Macadamia* has relatively strict requirements for growth conditions and is currently concentrated in a very limited area within China [24,25], which is one of the main reasons for the relatively low genetic diversity. Meanwhile, evolutionary processes involving short generation times and low mutation rates may also contribute to the moderate level of genetic diversity in *Macadamia* [26]. Finally, intensive human cultivation is another factor that reduces effective population size: cultivated macadamia is clonally propagated by grafting and, like many vegetatively propagated tree crops, carries the cumulative signature of founder events and selection during domestication [1,23]. Most of the individual samples used in this study are derived from a limited number of parental combinations and a narrow original cultivar base, resulting in a relatively narrow genetic foundation of the cultivated germplasm. Nevertheless, compared with previous SSR analyses of macadamia, the present study benefits from a larger number of individuals and more diverse source populations, thus providing more comprehensive representativeness of cultivated macadamia germplasm in South China.

Generally, when *F*_ST_ > 0.25, the level of genetic differentiation among populations is considered high [27]. The analysis of molecular variance (AMOVA) among macadamia populations yielded an *F*_ST_ of 0.304, although the majority of the genetic variation resided within populations. This predominance of within population variation is a recurrent pattern in long-lived, perennial, outcrossing plants [21]. *Macadamia* is a typical perennial evergreen tree and a predominantly outcrossing, partially self-incompatible species; these biological characteristics underpin its breeding and cultivation management [21,28,29]. The relative migration analysis using divMigrate revealed asymmetric relative migration patterns among populations, with bidirectional relative migration rates exceeding 0.7 in several pairwise comparisons. However, it should be cautioned that divMigrate estimates relative migration based on genetic differentiation rather than direct contemporary gene flow, and these values should not be interpreted as direct estimates of dispersal rates [13]. *Macadamia* is an animal and insect pollinated (zoophilous/entomophilous) tree to which wind contributes negligibly, *Macadamia* produces pollen grains that are large and heavy, with airborne dispersal typically not exceeding 10 m; even with supplementary insect pollination, the effective pollination distance is only 50–100 m, and fruit set declines significantly [23,27,29]. Consequently, long-distance genetic exchange with other populations is difficult. Moreover, *Macadamia* seeds are characterized by high density, heavy weight, and a hard woody endocarp. Under natural conditions, seeds are dispersed within the crown projection area of the mother tree, which greatly limits population expansion [23,30,31]. Deviations from HWE were observed at multiple locus-by-population comparisons; after false discovery rate (FDR, Benjamini–Hochberg) correction, 13 significant comparisons remained, potentially reflecting null alleles, Wahlund effects, or the small within-population sample sizes characteristic of cultivated germplasm collections. To assess the impact of null alleles, *F*_ST_ was recalculated using the ENA (excluding null alleles) method in FreeNA; the global *F*_ST_ without ENA correction (0.0351) was nearly identical to the ENA-corrected value (0.0436), with an absolute difference of only 0.0085, indicating a negligible effect of null alleles on differentiation estimates. Linkage disequilibrium (LD) analyses using Genepop v4.8.6 revealed that among 58 FDR-significant locus pairs, 49 (84.5%) involved loci on different chromosomes and only 9 (15.5%) involved loci on the same chromosome, suggesting that the observed LD was largely attributable to demographic effects rather than physical linkage; all 18 loci were therefore retained for downstream analyses.

Genetic diversity is an important indicator for the conservation and management of plant genetic resources. In this study, the polymorphism information content (PIC) values of macadamia populations ranged from 0.079 to 0.752, with a mean of 0.345. This is consistent with the genetic level previously reported for cultivated macadamia germplasm in Brazil [32], but slightly lower than the PIC levels reported for wild macadamia germplasm by Mai et al. (2020) and the PIC values of genomic SSRs reported by Wang et al. (2022) [14,18]. These results indicate that the 104 cultivated individuals analyzed in this study maintain a moderate level of genetic diversity, which may be attributed to small population sizes and founder effects characteristic of cultivated germplasm [33]. Discrepancies in PIC compared with wild germplasm could arise from differences in the type and number of genetic markers used and in the number of scored microsatellite alleles, or from variations in population and sample sizes, all of which are known to influence SSR-derived diversity and differentiation estimates [17,21]. Geographic distribution range, habitat type, and species characteristics can significantly affect population structure and genetic diversity [34,35]. Based on STRUCTURE analysis and principal coordinates analysis (PCoA), the 15 populations in this study could be clearly divided into two genetic groups, with the CA population forming an independent genetic cluster. The distinctiveness of CA is primarily due to its elite germplasm origin, with male parent 900 and female parent 695; the male parent 900 is a later-introduced Australian cultivar with an independent genetic background, which leads to its separation as a distinct lineage in genetic clustering. Regarding outlier loci, the candidate locus MacChr13-2 was detected by Arlequin’s hierarchical island model but not by BayeScan (FDR = 0.05). Such between-method discordance is expected, because *F*_ST_-outlier scans are sensitive to the underlying demographic history and population structure and depend strongly on the number and genomic coverage of the markers examined [36]. Given that only 18 loci were used, and that outlier detection with so small a marker panel is statistically underpowered, this putative signal should be interpreted with caution; the locus may be linked to a genomic region influenced by artificial selection during domestication or by demographic processes, but further validation with a larger, genome-wide marker panel is required [10,36]. It should be acknowledged that the 18 gSSR loci employed in this study provide sufficient power for assessing overall genetic diversity and broad population differentiation, but have limited resolution for fine-scale inference of population structure (K), precise migration estimates, and robust outlier detection under selection.

Based on the findings on genetic diversity and population structure of the cultivated *Macadamia* germplasm, we propose the following recommendations for germplasm management and utilization. Populations CK and CM exhibited the highest genetic diversity (H_e_, N_e_, I, and private alleles) and should be prioritized for germplasm maintenance and serve as core parental material for breeding programs. Population CA, while not the most genetically diverse, is recognized for its genetic distinctiveness and should be retained in the germplasm collection as a source of unique parental material reflecting Australian cultivar backgrounds. Populations with relatively low diversity (e.g., CA, CI, CF) may benefit from germplasm augmentation through controlled crosses with more diverse accessions to enhance their adaptive potential. These recommendations pertain exclusively to the management and utilization of cultivated *Macadamia* germplasm and do not extend to the conservation of wild populations.

## 5. Conclusions

In conclusion, the 104 cultivated *Macadamia* germplasm accessions examined in this study maintain moderate genetic diversity (H_e_ = 0.4814) but exhibit pronounced population differentiation (*F*_ST_ = 0.304). Two distinct genetic groups were resolved, with the CA population forming an independent lineage owing to its unique parental background rather than high genetic diversity. The relative migration patterns and biological characteristics of macadamia (limited pollen and seed dispersal) contribute to the observed genetic architecture, while human-mediated cultivation has further shaped the diversity of the cultivated germplasm. For germplasm management and breeding, we recommend prioritizing the most genetically diverse populations (CK and CM) for core collection maintenance and parental selection, retaining CA as a source of unique germplasm, and augmenting low diversity populations through controlled crosses. These findings provide a foundation for the informed management and utilization of cultivated macadamia germplasm in South China.

## Figures and Tables

**Figure 1:**
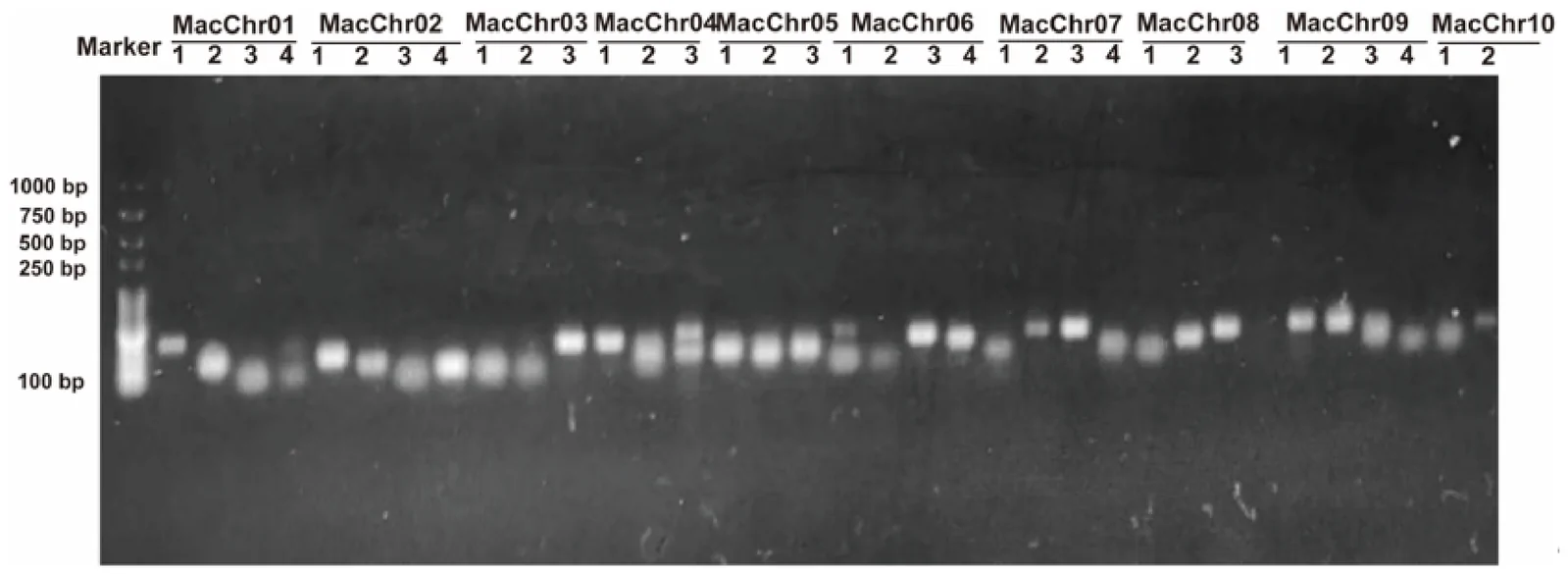
Agarose-gel electrophoresis results of PCR amplification using partial primers.

**Figure 2:**
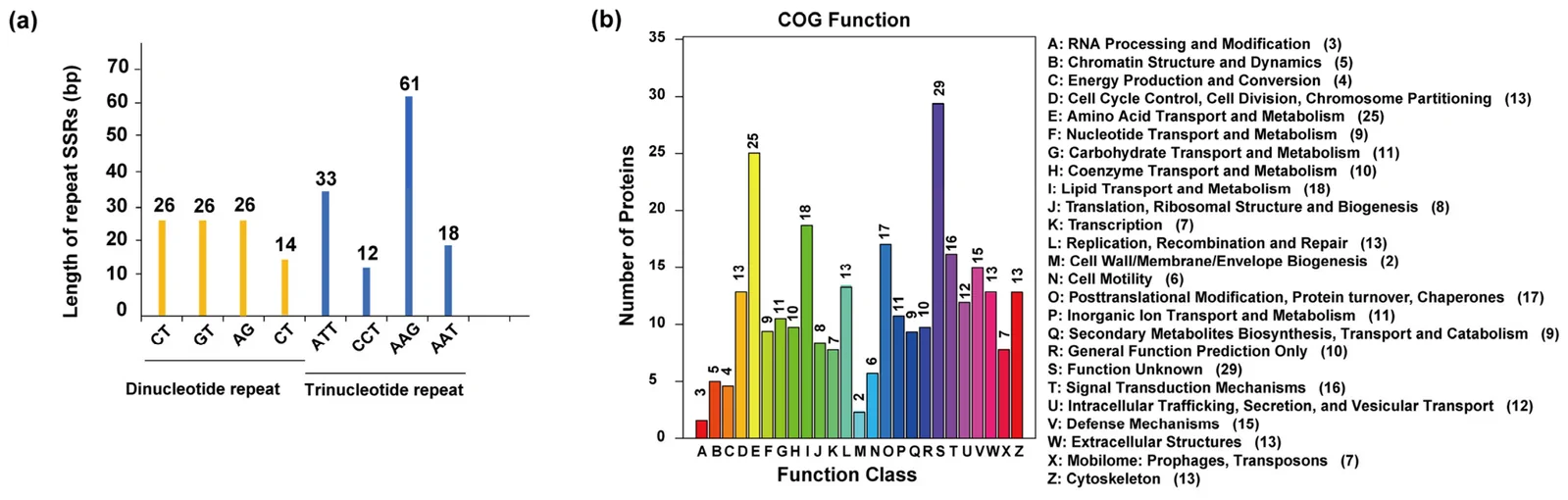
Characterization of SSR motif types and KOG functional annotation: (**a**) distribution and proportions of repeat motif types among identified SSR loci; (**b**) KOG functional classification of SSR-containing sequences.

**Figure 3:**
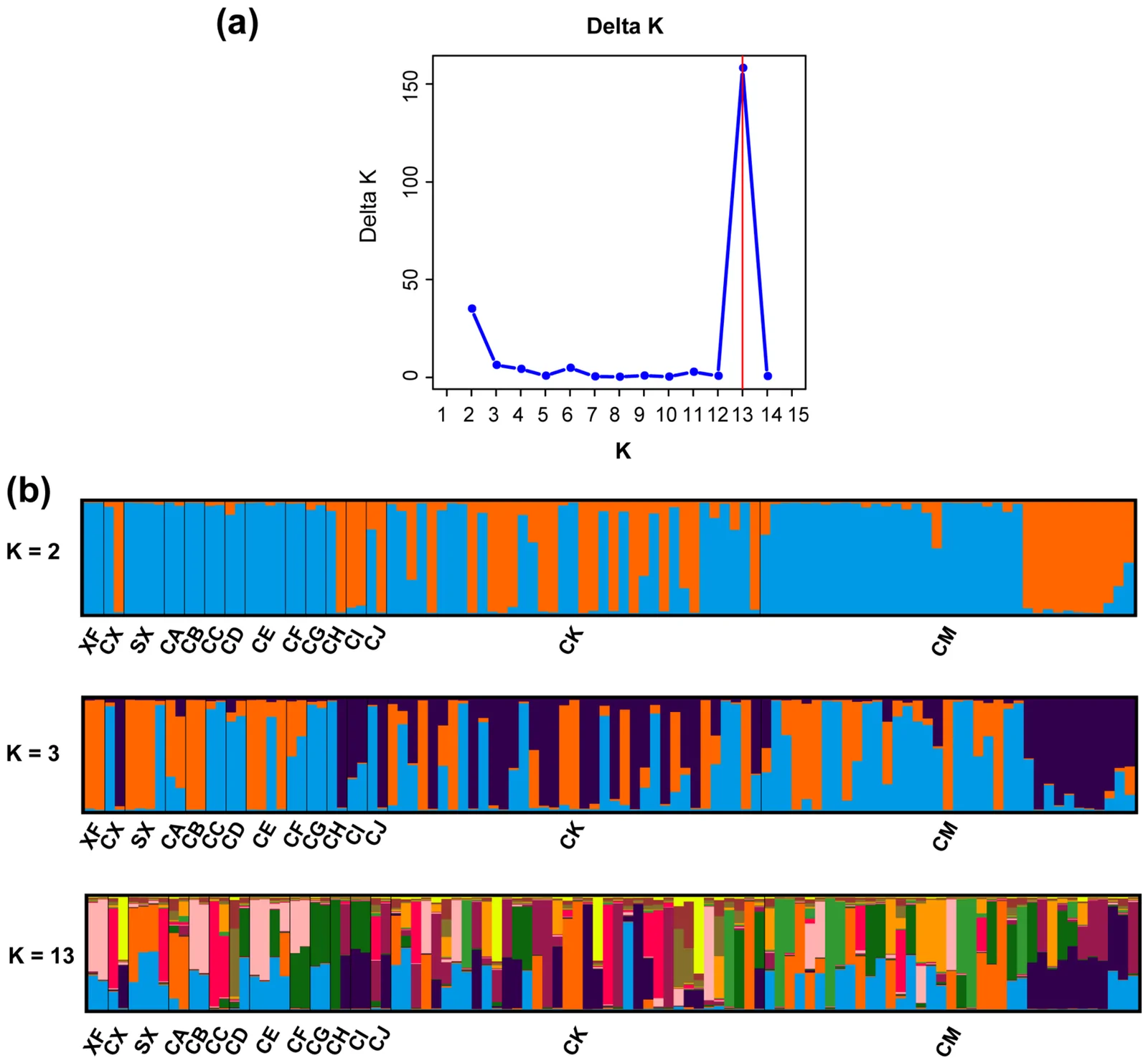
The genetic structure of the 15 populations: (**a**) estimation of population structure using delta K (ΔK) with the number of clusters ranging from 1 to 15; (**b**) estimation of population structure using the STRUCTURE analysis.

**Figure 4:**
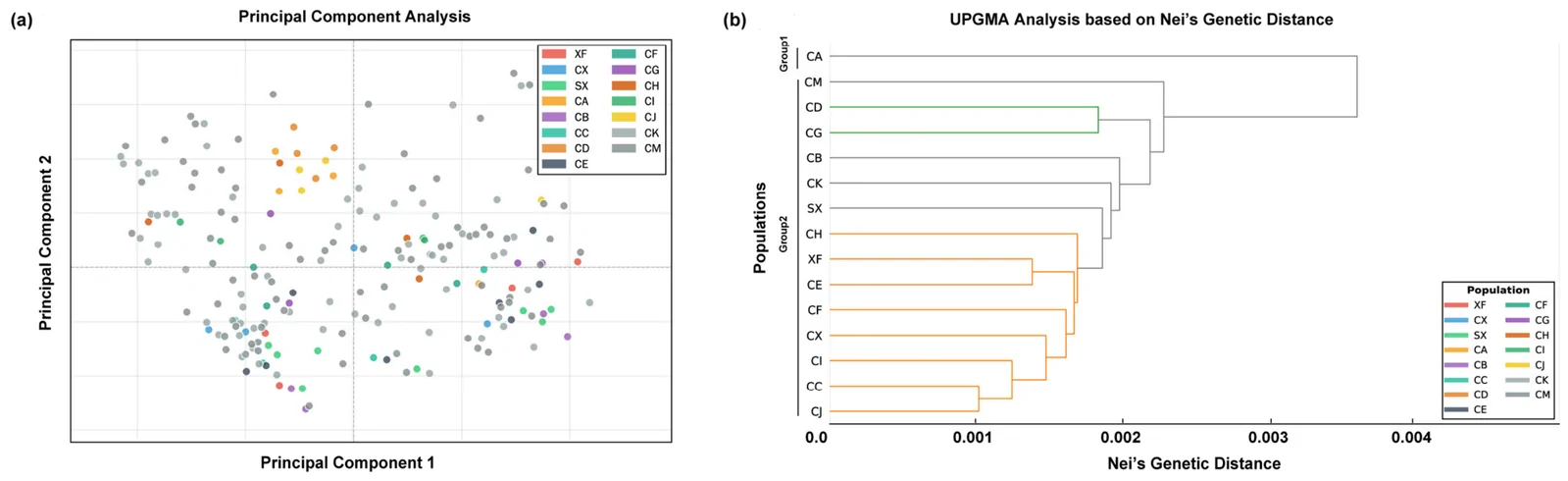
The Genetic variation and relationships among the *Macadamia* populations: (**a**) principal coordinate analysis (PCoA) based on pairwise genetic distance; (**b**) UPGMA dendrogram based on Nei’s genetic distance.

**Figure 5:**
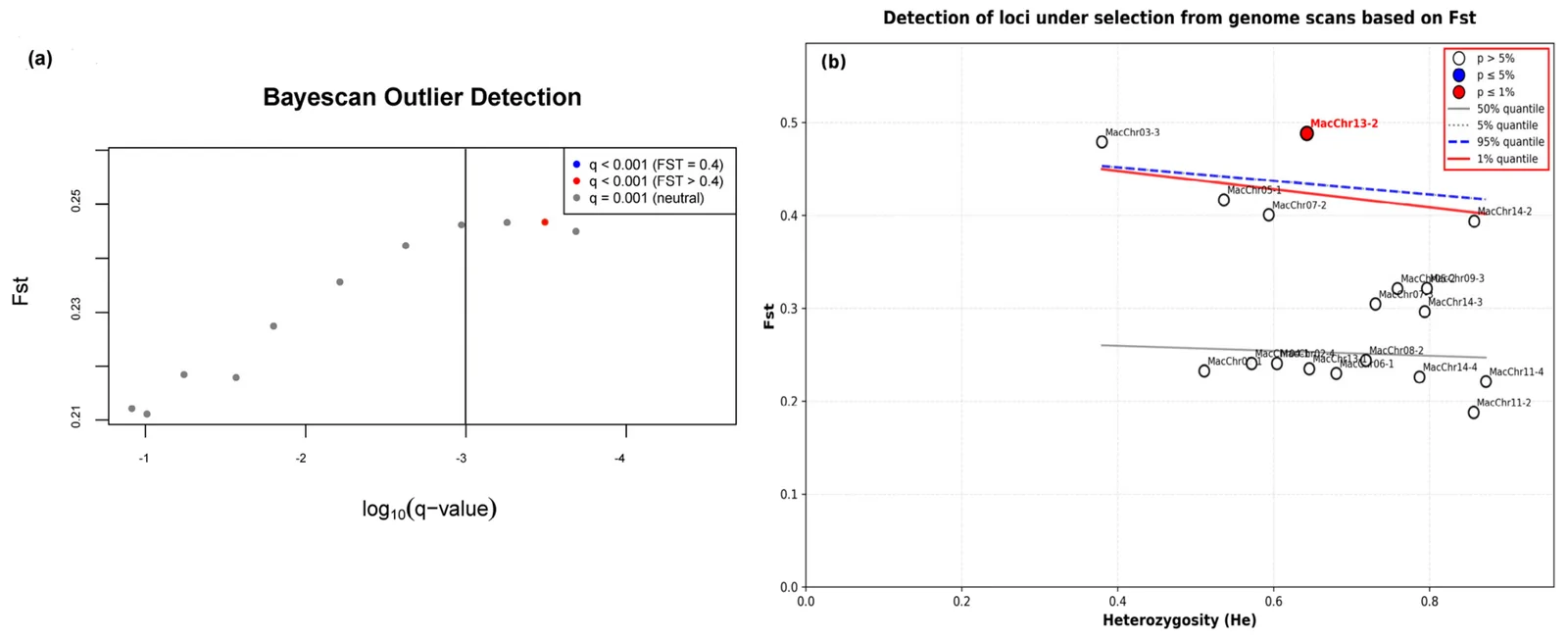
Identification of loci under outlier distribution: (**a**) outlier loci detected by BayeScan; (**b**) outlier loci detected by the hierarchical island model implemented in Arlequin.

**Table 1:** PCR amplification system used in this study.

Component	Volume (µL)
Buffer (10 × containing Mg^2+^)	1 µL
dNTPs (2.5 mM each)	0.4 µL
Taq (5 U/µL)	0.05 µL
Forward primer (10 mM)	0.25 µL
Reverse primer (10 mM)	0.25 µL
Template DNA (15 ng/µL)	2.0 µL
ddH_2_O	6.05 µL
Total	10.0 µL

**Table 2:** Genetic diversity analysis of 15 *Macadamia* populations.

N	Pop	N_a_	N_e_	I	H_o_	H_e_	N_P_	A_r_	F_IS_
X	XF	2.167	1.978	0.669	0.694	0.438	0	2.311	0.518
CX	CX	2.385	2.130	0.734	0.556	0.451	0	2.803	0.211
SX	SX	2.944	2.252	0.836	0.583	0.479	1	1.538	0.189
CA	CA	2.111	1.904	0.611	0.556	0.389	1	2.417	0.318
CB	CB	2.167	2.019	0.657	0.697	0.424	0	2.318	0.529
CC	CC	2.167	1.945	0.657	0.639	0.424	2	1.510	0.404
CD	CD	2.444	2.215	0.758	0.583	0.465	1	2.304	0.229
CE	CE	2.778	2.097	0.816	0.639	0.491	0	1.536	0.243
CF	CF	2.111	1.967	0.645	0.639	0.424	0	2.219	−0.426
CG	CG	2.278	2.085	0.719	0.639	0.465	0	1.500	0.344
CH	CH	2.667	2.445	0.888	0.667	0.549	0	2.487	0.215
CI	CI	2.056	1.922	0.609	0.694	0.395	0	1.636	0.551
CJ	CJ	2.333	2.152	0.722	0.694	0.451	1	2.775	−0.455
CK	CK	7.167	4.141	1.499	0.679	0.699	53	2.492	0.038
CM	CM	5.889	3.555	1.380	0.632	0.677	10	2.284	0.073

Note: N_a_, mean number of alleles per locus; N_e_, number of effective alleles; I, Shannon’s information index; H_o_, observed heterozygosity; H_e_, expected heterozygosity; N_p_, number of private alleles; A_r_, allelic richness; F**_IS_**, inbreeding coefficient.

**Table 3:** The result of Hardy–Weinberg test for 15 *Macadamia* populations.

SSR Locus	XF	CX	SX	CA	CB	CC	CD	CE	CF	CG	CH	CI	CJ	CK	CM	Total
Mac001	ns	ns	**	***	ns	-	ns	-	***	-	ns	-	-	ns	-	***
Mac002	-	ns	ns	-	ns	ns	-	ns	***	ns	ns	***	ns	-	**	***
Mac003	***	ns	**	ns	-	ns	**	ns	**	-	ns	-	ns	ns	ns	**
Mac004	ns	***	ns	-	ns	**	ns	ns	ns	***	ns	ns	ns	ns	-	***
Mac005	ns	ns	ns	ns	-	ns	ns	**	ns	-	**	ns	-	ns	***	**
Mac006	ns	**	-	ns	***	ns	**	ns	-	***	**	**	ns	***	ns	***
Mac007	ns	ns	**	**	**	ns	-	-	**	-	***	**	ns	-	ns	**
Mac008	-	ns	***	ns	**	ns	ns	***	ns	**	-	***	**	ns	***	***
Mac009	ns	***	-	**	***	-	ns	ns	***	**	ns	ns	***	-	ns	***
Mac010	-	ns	ns	***	-	-	***	ns	**	ns	-	***	ns	ns	ns	***
Mac011	ns	***	ns	ns	ns	ns	**	ns	ns	***	ns	ns	**	ns	ns	**
Mac012	ns	ns	**	ns	ns	ns	ns	ns	ns	-	ns	**	ns	***	ns	***
Mac013	***	ns	-	ns	ns	ns	**	ns	-	-	-	ns	**	**	-	***
Mac014	ns	ns	ns	ns	ns	-	ns	-	ns	**	ns	**	ns	ns	ns	**
Mac015	-	**	ns	-	ns	ns	**	ns	ns	ns	***	ns	***	ns	ns	***
Mac016	ns	-	-	ns	**	-	ns	ns	ns	ns	***	ns	-	-	ns	***
Mac017	ns	-	ns	**	ns	***	ns	ns	-	**	***	ns	-	**	**	***
Mac018	-	**	-	***	ns	-	**	-	**	ns	ns	**	ns	ns	ns	***

Note: ns = not significant, ** *p* < 0.01, *** *p* < 0.001.

**Table 4:** Analysis of molecular variance (AMOVA) of *Macadamia* populations.

Source of Variation	d*f*	Sum of Squares	Variance Components		Percentage of Variation (%)
AMOVA					
Among Populations	14	543.3943	0.840066		16.39
Within Populations	89	762.8649	4.285758		83.61
Total	103	1306.2592		5.125823	
Fixation Index		*F*_ST_ = 0.304; *p* < 0.01			

Note: *F*_ST_, genetic differentiation among populations within groups.

**Table 5:** Estimated pairwise migration rates among *Macadamia* populations.

Populations	CA	CB	CC	CD	CE	CF	CG	CH	CI	CJ	CK	CM	CX	SX	XF
CA	1	0.024391	0.041399	0.02661	0.031511	0.029318	0.029697	0.048729	0.037949	0.035496	0.072011	0.059141	0.052457	0.070083	0.028835
CB	0.024391	1	0.032332	0.033061	0.062859	0.033223	0.037577	0.033700	0.026819	0.036315	0.047763	0.054703	0.038655	0.048319	0.112686
CC	0.041399	0.032332	1	0.033586	0.031469	0.051325	0.02579	0.086787	0.036168	0.066529	0.092726	0.062991	0.097436	0.056386	0.024048
CD	0.026612	0.033061	0.033586	1	0.030147	0.024021	0.044221	0.031535	0.033402	0.098526	0.047349	0.051363	0.028218	0.075109	0.022829
CE	0.031511	0.062859	0.031469	0.030147	1	0.033525	0.029418	0.036526	0.027104	0.038129	0.056121	0.071414	0.039159	0.057503	0.085506
CF	0.029318	0.033223	0.051325	0.024021	0.033525	1	0.025614	0.075004	0.023693	0.036855	0.084662	0.063223	0.082168	0.034762	0.030624
CG	0.029697	0.037577	0.02579	0.044221	0.029418	0.025614	1	0.025629	0.037303	0.038323	0.043207	0.048759	0.02446	0.079339	0.03531
CH	0.048729	0.033700	0.086787	0.031535	0.036526	0.075004	0.025629	1	0.03032	0.04332	0.158114	0.083517	0.119473	0.049519	0.030381
CI	0.037949	0.026819	0.036168	0.033402	0.027104	0.023693	0.037303	0.03032	1	0.041748	0.064987	0.08325	0.044045	0.040796	0.02458
CJ	0.035496	0.036315	0.066529	0.098526	0.038129	0.036855	0.038323	0.04332	0.041748	1	0.087373	0.08915	0.051032	0.071452	0.026356
CK	0.072011	0.047763	0.092726	0.047349	0.056121	0.084662	0.043207	0.158114	0.064987	0.087373	1	0.452788	0.159804	0.094908	0.044536
CM	0.059141	0.054703	0.062991	0.051363	0.071414	0.063223	0.048759	0.083517	0.08325	0.08915	0.452788	1	0.12357	0.092192	0.046418
CX	0.052457	0.038655	0.097436	0.028218	0.039159	0.082168	0.02446	0.119473	0.044045	0.051032	0.159804	0.12357	1	0.04314	0.031141
SX	0.070083	0.048319	0.056386	0.075109	0.057503	0.034762	0.079339	0.049519	0.040796	0.071452	0.094908	0.092192	0.04314	1	0.040574
XF	0.028835	0.112686	0.024048	0.022829	0.085506	0.030624	0.03531	0.030381	0.02458	0.026356	0.044536	0.046418	0.031141	0.040574	1

## Data Availability

The raw data supporting the conclusions of this article are included in the Supplementary Materials. The data are not publicly available due to institutional restrictions.

## Supplementary Materials

The following supporting information can be downloaded at https://www.mdpi.com/article/10.3390/life16101618/s1: Table S1: Sampling information and cross combinations of *Macadamia* accessions. Table S2: Information of the SSR primer pairs developed in this study. Table S3: The KOG annotation of 18 SSR locus. Table S4: Allele sizes (bp) at 18 SSR loci for 104 *Macadamia* individuals.

## References

[1] Nybom H., Lācis G. (2021). Recent Large-Scale Genotyping and Phenotyping of Plant Genetic Resources of Vegetatively Propagated Crops. Plants.

[2] Sun L., Lai M., Ghouri F., Nawaz M.A., Ali F., Baloch F.S., Nadeem M.A., Aasim M., Shahid M.Q. (2024). Modern Plant Breeding Techniques in Crop Improvement and Genetic Diversity: From Molecular Markers and Gene Editing to Artificial Intelligence—A Critical Review. Plants.

[3] Gao C., Chen C., Liu N., Liu F., Su X., Liu C., Huang Q. (2024). Genetic Diversity and Association Analysis of Traits Related to Water-Use Efficiency and Nitrogen-Use Efficiency of Populus deltoides Based on SSR Markers. Int. J. Mol. Sci..

[4] Geethanjali S., Kadirvel P., Anumalla M., Hemanth Sadhana N., Annamalai A., Ali J. (2024). Streamlining of Simple Sequence Repeat Data Mining Methodologies and Pipelines for Crop Scanning. Plants.

[5] Varshney R.K., Graner A., Sorrells M.E. (2005). Genic microsatellite markers in plants: Features and applications. Trends Biotechnol..

[6] Singh J., Sharma A., Sharma V., Gaikwad P.N., Sidhu G.S., Kaur G., Kaur N., Jindal T., Chhuneja P., Rattanpal H.S. (2023). Comprehensive genome-wide identification and transferability of chromosome-specific highly variable microsatellite markers from citrus species. Sci. Rep..

[7] Turudi A., Liber Z., Grdia M., Jake J., Varga F., Poljak I., Atovi Z. (2024). Dig-up Primers: A Pipeline for Identification of Polymorphic Microsatellites Loci within Assemblies of Related Species. Int. J. Mol. Sci..

[8] Topp B.L., Nock C.J., Hardner C.M., Alam M., O’Connor K.M. (2019). Advances in Plant Breeding Strategies: Nut and Beverage Crops.

[9] Dhakal Poudel P., Cowan M., Shaw L., De Faveri J., Topp B., Alam M. (2023). Macadamia Breeding for Reduced Plant Vigor: Progress and Prospects for Profitable and Sustainable Orchard Systems. Sustainability.

[10] Lin J., Zhang W., Zhang X., Ma X., Zhang S., Chen S., Wang Y., Jia H., Liao Z., Lin J. (2022). Signatures of selection in recently domesticated macadamia. Nat. Commun..

[11] Niu Y.F., Li G., Ni S.B., He X., Zheng C., Liu Z., Gong L., Kong G., Li W., Liu J. (2022). The Chromosome-Scale Reference Genome of Macadamia tetraphylla Provides Insights Into Fatty Acid Biosynthesis. Front. Genet..

[12] Spain C.S., Lowe A.J. (2011). Genetic consequences of subtropical rainforest fragmentation on *Macadamia tetraphylla* (Proteaceae). Silvae Genet..

[13] O’Connor K., Powell M., Nock C., Shapcott A. (2015). Crop to wild gene flow and genetic diversity in a vulnerable Macadamia (Proteaceae) species in New South Wales, Australia. Biol. Conserv..

[14] Mai T., Alam M., Hardner C., Henry R., Topp B. (2020). Genetic Structure of Wild Germplasm of Macadamia: Species Assignment, Diversity and Phylogeographic Relationships. Plants.

[15] O’Connor K., Kilian A., Hayes B., Hardner C., Nock C., Baten A., Alam M., Topp B. (2019). Population structure, genetic diversity and linkage disequilibrium in a macadamia breeding population using SNP and silicoDArT markers. Tree Genet. Genomes.

[16] Ranketse M., Hefer C.A., Pierneef R., Fourie G., Myburg A.A. (2022). Genetic diversity and population structure analysis reveals the unique genetic composition of South African selected macadamia accessions. Tree Genet. Genomes.

[17] Tong Y.W., Lewis B.J., Zhou W.M., Mao C.R., Wang Y., Zhou L., Yu D.P., Dai L.M., Qi L. (2019). Genetic Diversity and Population Structure of Natural Pinus koraiensis Populations. Forests.

[18] Wang P., Mo Y., Wang Y., Fei Y., Huang J., Ni J., Xu Z.F. (2022). Macadamia germplasm and genomic database (MacadamiaGGD): A comprehensive platform for germplasm innovation and functional genomics in Macadamia. Front. Plant Sci..

[19] Yuan R., Zhang N., Zhang Q. (2024). The impact of habitat loss and fragmentation on biodiversity in global protected areas. Sci. Total Environ..

[20] Nock C.J., Elphinstone M.S., Ablett G., Kawamata A., Hancock W., Hardner C.M., King G.J. (2014). Whole Genome Shotgun Sequences for Microsatellite Discovery and Application in Cultivated and Wild Macadamia (Proteaceae). Appl. Plant Sci..

[21] Hilde N. (2004). Comparison of different nuclear DNA markers for estimating intraspecific genetic diversity in plants. Mol. Ecol..

[22] Zhou Q., Mu K., Ni Z., Liu X., Li Y., Xu L.-a. (2020). Analysis of genetic diversity of ancient Ginkgo populations using SSR markers. Ind. Crops Prod..

[23] Hardner C.M., Peace C., Lowe A.J., Neal J., Pisanu P. (2009). Genetic resources and domestication of Macadamia. Hortic. Rev..

[24] Hardner C.M., Wall M., Cho A. (2019). Global Macadamia Science: Overview of the Special Section. HortScience.

[25] Allan P. (2016). Development of the macadamia nut industry in South Africa. Subtrop. Q. J..

[26] Hewitt G. (2000). The genetic legacy of the Quaternary ice ages. Nature.

[27] Wright S.W. (1965). The Interpretation of Population Structure by F-Statistics with Special Regard to Systems of Mating. Evolution.

[28] Trueman S.J. (2013). The reproductive biology of macadamia. Sci. Hortic..

[29] Howlett B.G., Nelson W.R., Pattemore D.E., Gee M. (2015). Pollination of macadamia: Review and opportunities for improving yields. Sci. Hortic..

[30] Graham C.D., Warneke C.R., Weber M., Brudvig L.A. (2022). The impact of habitat fragmentation on domatia-dwelling mites and a mite-plant-fungus tritrophic interaction. Landsc. Ecol..

[31] Mast A.R., Willis C.L., Jones E.H., Downs K.M., Weston P.H. (2008). A smaller Macadamia from a more vagile tribe: Inference of phylogenetic relationships, divergence times, and diaspore evolution in Macadamia and relatives (tribe Macadamieae; Proteaceae). Am. J. Bot..

[32] Pereira-Lorenzo S., Urrestarazu J., Ramos-Cabrer A.M., Miranda C., Pina A., Dapena E., Moreno M.A., Errea P., Llamero N., Díaz-Hernández M.B. (2017). Analysis of the genetic diversity and structure of the Spanish apple genetic resources suggests the existence of an Iberian genepool. Ann. Appl. Biol..

[33] Wang C.B., Wang T., Su Y.J. (2014). Phylogeography of *Cephalotaxus oliveri* (Cephalotaxaceae) in relation to habitat heterogeneity, physical barriers and the uplift of the Yungui Plateau. Mol. Phylogenetics Evol..

[34] Jia H., Yang H., Sun P., Li J., Zhang J., Guo Y., Han X., Zhang G., Lu M., Hu J. (2016). De novo transcriptome assembly, development of EST-SSR markers and population genetic analyses for the desert biomass willow, *Salix psammophila*. Sci. Rep..

[35] Li X.-Y., Lin X.-Y., Ruhsam M., Chen L., Wu X.-T., Wang M.-q., Thomas P.I., Wen Y.-F. (2019). Development of microsatellite markers for the critically endangered conifer *Glyptostrobus pensilis* (Cupressaceae) using transcriptome data. Silvae Genet..

[36] Luikart G., England P.R., Tallmon D., Jordan S., Taberlet P. (2003). The power and promise of population genomics: From genotyping to genome typing. Nat. Rev. Genet..

