# Supplementary material for: Development and Application of gSSRs Markers in Macadamia from Genomic Sequences
Source: Life (Basel). 2026 Sep 26;16(10):1618. doi: 10.3390/life16101618 (PMC13705751; doi:10.3390/life16101618)
Supplement: Supplementary file 1 [file life-16-01618-s001.zip › life-4458497-supplementary.pdf]

Supplementary Table S1. Sampling information and cross combinations of *Macadamia* accessions.

Sample No.	Cross combination (M×F)	Male parent	Female parent	Population abbreviations	Sowing method	Year of establishment
1	900×695	123	115	XF1	Sandy soil, flat	2021
2	695×A16	115	113	CM	Sandy soil, flat	2021
3	OC×A16	126	113	CM	Sandy soil, flat	2021
4	Gui1×OV	127	125	CM	Sandy soil, flat	2021
5	696×OC	696	126	CM	Sandy soil, flat	2021
6	NanYa1×695	111	115	CX3	Sandy soil, flat	2021
7	Gui1×695	127	115	SX8	Sandy soil, flat	2021
8	NanYa1×Gui1	111	127	CA9	Sandy soil, flat	2021
9	JW×900	108	123	CM	Sandy soil, flat	2021
10	JW×Ziyou May	108	Ziyou May	CK31	Sandy soil, flat	2021
11	900×Gui1	123	127	CB11	Sandy soil, flat	2021
12	900×A16	123	113	CM	Sandy soil, flat	2021
13	OV×695	125	115	CM	Sandy soil, flat	2021
14	695×OV	115	125	CM	Sandy soil, flat	2021
15	695×A4	115	124	CM	Sandy soil, flat	2021
16	A16×Ziyou	113Ziyou	—	CK32	Sandy soil, flat	2021
17	Gui1×NanYa3	127	119	CM	Sandy soil, flat	2021
18	900×Gui1	123	127	CB12	Sandy soil, flat	2021
19	OV×Gui1	125	127	CM	Sandy soil, flat	2021
20	NanYa3×Dapeng	119Dapeng	—	CK33	Sandy soil, flat	2021
21	NanYa1×A16	111	113	CC13	Sandy soil, flat	2021
22	NanYa1×Gui1	NanYa1	Gui1	CK34	Sandy soil, flat	2021
23	OC×695	126	115	CD15	Sandy soil, flat	2021
24	695×OC	115	126	CM	Sandy soil, flat	2021
25	OC×695	126	115	CD16	Sandy soil, flat	2021
26	Dapeng109	Dapeng109	—	CK35	Sandy soil, flat	2021
27	A16×Gui1	113	127	CM	Sandy soil, flat	2021
28	695×900	115	123	CE17	Sandy soil, flat	2021
29	695×Gui1	115	127	CE19	Sandy soil, flat	2021
30	NanYa1×Ziyou	111Ziyou	—	CK36	Sandy soil, flat	2021
31	NanYa1×Gui1	111	127	CA10	Sandy soil, flat	2021
32	NanYa1×A4	111	124	CM	Sandy soil, flat	2021
33	OC×A4	126	124	CM	Sandy soil, flat	2021
34	Gui1×695	127	115	SX7	Sandy soil, flat	2021
35	900Ziyou	123Ziyou	—	CK37	Sandy soil, flat	2021
36	A16×900	113	123	CM	Sandy soil, flat	2021
37	900×OC	123	126	CF21	Sandy soil, flat	2021
38	A16×OV	113	125	CM	Sandy soil, flat	2021
39	OV×Ziyou	125Ziyou	—	CK38	Sandy soil, flat	2021
40	900×OC	123	126	CF22	Sandy soil, flat	2021
41	A16695	113115	—	CM	Sandy soil, flat	2021
42	900×695	123	115	XF2	Sandy soil, flat	2021
43	Gui1×695	127	115	SX5	Sandy soil, flat	2021
44	Gui1×695	127	115	SX6	Sandy soil, flat	2021
45	Gui1×900	127	123	CM	Sandy soil, flat	2021
46	695×900	115	123	CE18	Sandy soil, flat	2021
47	NanYa12×Dapeng	1112Dapeng	—	CK39	Sandy soil, flat	2021
48	OV×900	125	123	CM	Sandy soil, flat	2021
49	NanYa1×OC	111	126	CM	Sandy soil, flat	2021
50	OC×695	126	115	CG23	Sandy soil, flat	2021
51	OC×900	126	123	CM	Sandy soil, flat	2021
52	OC×695	126	115	CG24	Sandy soil, flat	2021
53	695	115	—	CK40	Sandy soil, flat	2021

Sample No.	Cross combination (M×F)	Male parent	Female parent	Population abbreviations	Sowing method	Year of establishment
54	OC PC	126 PC	—	CK	Sandy soil, flat	2021
55	Gui1×JW	127	108	CM	Sandy soil, flat	2021
56	OC×Gui1	126	127	CM	Sandy soil, flat	2021
57	NanYa1×A16	111	113	CC14	Sandy soil, flat	2021
58	695×JW	115	108	CM	Sandy soil, flat	2021
59	NanYa3Ziyou	119Ziyou	—	CK41	Sandy soil, flat	2021
60	H2Ziyou	110Ziyou	—	CK	Sandy soil, flat	2021
61	Nan3×Nan2	119	117	CH25	Sandy soil, flat	2021
62	OC×922	126	922	CM	Sandy soil, flat	2021
63	Nan12×Nan3	116	119	CM	Sandy soil, flat	2021
64	OC×Ziyou May	126Ziyou	—	CK	Sandy soil, flat	2021
65	OC×Ziyou May	126Ziyou	—	CK	Sandy soil, flat	2021
66	Nan12×Nan2	116	117	CM	Sandy soil, flat	2021
67	OC×H2	126	110	CI27	Sandy soil, flat	2021
68	Nan3×H2	119	110	CM	Sandy soil, flat	2021
69	H2×Nan12	110	116	CM	Sandy soil, flat	2021
70	922×H2	922	110	CM	Sandy soil, flat	2021
71	H2×922	110	922	CM	Sandy soil, flat	2021
72	Nan3×334	119	118	CM	Sandy soil, flat	2021
73	H2×695	110	115	CM	Sandy soil, flat	2021
74	Nan3×Nan12	119	116	CJ29	Sandy soil, flat	2021
75	Nan3×Nan2	119	117	CH26	Sandy soil, flat	2021
76	695×H2	115	110	CM	Sandy soil, flat	2021
77	695×922	115	922	CM	Sandy soil, flat	2021
78	Nan1× 695	111	115	CX4	Sandy soil, flat	2021
79	OC× H2	126	110	CI28	Sandy soil, flat	2021
80	Nan3×Nan12	119	116	CJ30	Sandy soil, flat	2021
81	695×Gui1	115	127	CE20	Sandy soil, flat	2021
82	Nan3×Ziyou	119	Ziyou	CK	Sandy soil, flat	2021
83	108Pengnei	108	Pengnei	CK	Sandy soil, flat	2021
84	109Pengnei	109	Pengnei	CK	Sandy soil, flat	2021
85	JW	108	—	CK	Sandy soil, flat	2021
86	Gui	127	—	CK	Sandy soil, flat	2021
87	H2	110	—	CK	Sandy soil, flat	2021
88	NanYa1	111	—	CK	Sandy soil, flat	2021
89	788	112	—	CK	Sandy soil, flat	2021
90	788	112	—	CM	Sandy soil, flat	2021
91	A16	113	—	CK	Sandy soil, flat	2021
92	695	115	—	CK	Sandy soil, flat	2021
93	NanYa12	116	—	CK	Sandy soil, flat	2021
94	NanYa2	117	—	CK	Sandy soil, flat	2021
95	334	118	—	CK	Sandy soil, flat	2021
96	NanYa3	119	—	CK	Sandy soil, flat	2021
97	Dahong×NanYa3	Dahong	NanYa3	CK	Sandy soil, flat	2021
98	24×Pengnei	24	Pengnei	CK	Sandy soil, flat	2021
99	116×Pengnei	116	Pengnei	CK	Sandy soil, flat	2021
100	900	123	—	CK	Sandy soil, flat	2021
101	A4	124	—	CK	Sandy soil, flat	2021
102	OV	125	—	CK	Sandy soil, flat	2021
103	OC	126	—	CK	Sandy soil, flat	2021
104	Gui	127	—	CK	Sandy soil, flat	2021

Supplementary Table S2. Information of the SSR primer pairs developed in this study.

Primer name	Repeat motif	Mg^{2+} (mM)	Tm (℃)	size (bp)	Primer F (5'-3')	Primer R (5'-3')
MacChr01-1	(AAT)^6	2	60	382	AGCTAAGGCGAAAGGGTTATC	ACTCAGTGAGAAGGTCTTCAACGAT
MacChr01-2	(AAC)^6	2	60	203	CCGATGACGACGATGAAGTCT	CTTCCGCCGCTCCTCTATGGAT
MacChr01-3	(ATC)^8	2	60	284	TGATGAGGTTGGAATGGTAGT	AGGGAGATAAGAAATGACGGCAAA
MacChr01-4	(AAT)^9	2	60	273	TCCACATCATCATCTTGTCTT	TTGATTGGCTATCTTTCTCCAGACT
MacChr02-1	(ATC)^9	2	60	238	TCTCATCGTCCTCTTCCACCA	GCAGCAGTTGAGGCAGATCCA
MacChr02-2	(AAT)^7	2	60	164	GCCAGGTAACCAATTAGCATT	TGGGTCCTCCGCCAGATTTG
MacChr02-3	(AGG)^7	2	60	282	ATCCTGTGGACCGTTGATGTC	CGAACTGCTGGTTGCTGAGAC
MacChr02-4	(CTT)^8	2	60	127	AGGAGGATACCTGCTGGTTCT	TTGGGTTCTGAAGAGTGGGCTTTG
MacChr03-1	(AAT)^10	2	60	251	ACATACAAAGCCCTTCCCGAA	TTGCGGCGCTGTTTCTGGA
MacChr03-2	(AAT)^7	2	60	168	TTCAATCGAGTGCCACGCATT	TCCAGTCATCAGGGATGTTGTT
MacChr03-3	(AAG)^7	2	60	281	CATACGCTACGGAGGAGGA	CCAGTACGTCTAGCCATTATTC
MacChr04-1	(CGT)^8	2	60	292	TGCTCACCATCCTCGTCTACA	TTCACTCCACTAAGCGACTCCTC
MacChr04-2	(GTT)^9	2	60	181	TGAATTGGAAGTCACCGAATG	ACTCAGGAGCACCATTGTAACT
MacChr04-3	(GGT)^7	2	60	188	AGTACAGTGGAAGCCTGTTGA	GGGTGGTGGGTTTGTTTCTTAG
MacChr05-1	(ATT)^7	2	60	185	CTGATGTATGATGTGCTGGTG	AAGAGGAGGCAAGAAGACCAA
MacChr05-2	(AAG)^6	2	60	158	ACTGTCGGTACTGCCCAGAAA	TGTGTTCTCCAGTTGCCTGTGT
MacChr05-3	(GTT)^5	2	60	187	GCGGTTTAGGGCAAAGCAAAT	TGAGACAATGAGCAAGGATGGAGAC
MacChr06-1	(AGC)^7	2	60	322	GCCTCTCACAATGACCCAAGG	AGGTGTCTCTGCTTGAGTGGTT
MacChr06-2	(CCT)^8	2	60	190	CGGAGTGTCTTCACAGCATGG	GCGTCTGCCATTGGATCTTTGA
MacChr06-3	(AGC)^10	2	60	284	CCAGATTCTTCATTCATAGTC	TGGTGGTGTATAGGCTGTGTT
MacChr06-4	(CCT)^6	2	60	220	GATGCGATGCGGATGATGTC	GGTTCAGGAGGAGGAGAATGG
MacChr07-1	(CTT)^6	2	60	122	ATGTGCTCTACACTTGCCATG	CACTCAACACTGCTATCTTGCA
MacChr07-2	(ATT)^9	2	60	282	TAATACGATACGATGCTCTGA	AAGAACTGGTCACCGAACCTATA
MacChr07-3	(ACC)^6	2	60	269	GCAACAGTTGGCATGGAAGA	GGAGAGGCAGCGAAGTGAA
MacChr07-4	(AAG)^7	2	60	142	TTTCCACACCCAACCCACACC	GCAGGCGTAATGAACAGCTTGT
MacChr08-1	(AAT)^9	2	60	133	GGCTTCATCCACCTTACCATC	GGAGGGTGTGTTGGGTAATGCT
MacChr08-2	(AAC)^9	2	60	179	CCACGTAACGACTTCAATGCT	CCGTATGCACTAATCATCACCACTT
MacChr08-3	(ATT)^6	2	60	225	GGAACTTGGTTGACTGTCATG	CACAAGAACCGAAGCCTGGAAT
MacChr09-1	(ATT)^7	2	60	197	ACAGGGTTGGACATAGTAGGA	ACAGGGTTGGACATAGTAGGAAA
MacChr09-2	(CTT)^6	2	60	269	GCAGTAGATCAGTAACGCAGG	ACAGCAACAATGGTGTGCAATT
MacChr09-3	(ATT)^7	2	60	243	GGATGGGCTTTGTTGGATGGA	TGGAAGGGCTAAATGACTACCC
MacChr09-4	(GTT)^7	2	60	151	AGAGGGTGATCGGAATGGACA	TCGCAACCTCTACGATCTCTCC
MacChr10-1	(GAT)^9	2	60	229	TTGTTCACAAATCTGGCTAGT	TCAGCTCTACAAATCAGTCATCATG
MacChr10-2	(GGT)^10	2	60	173	ACTGGATGTTTGGTAGCCACG	CCCACTTGCCACACCTCTTTCT
MacChr10-3	(AAC)^6	2	60	217	TTCTAGCCTTTGAAACCACTC	CCCTTCCCTTTCCTTTCCATC
MacChr11-1	(ATT)^7	2	60	288	CCTCTCTGTAGGGCTCCTTGA	TGAGTGGAGGCAGGGTGTGA
MacChr11-2	(ATT)^14	2	60	232	CACACCATGTCACCTCTACCA	CAAGATGTATGAGACTTCCGAGAG
MacChr11-3	(GGT)^7	2	60	198	GCCTTAGCCAACCCGTACCAT	ACCACTACTACCACTGCCACCA
MacChr11-4	(AAG)^6	2	60	351	GCATGAGTGTAGCAGCAAGAG	ACTCCCTCACCACCCACCAA
MacChr12-1	(AAT)^7	2	60	194	CTTGAACCAACCAGAATCCAA	TTGCTCTCTATGTTTGGGTGTAAGG
MacChr12-2	(CTT)^7	2	60	217	GCCTTCTGCTCCGCACTCTT	GCCTTCTGCTCCGCACTCTT
MacChr12-3	(CTT)^8	2	60	261	ACCCGCCTGACCAATAAATGA	ATACTCCTATCCCTAACACCATCGC
MacChr13-1	(GTT)^7	2	60	138	AGTCGGTTCCATTTAGTTGGC	TGGGAGAAATCGTCAGCAAAGTAG
MacChr13-2	(AAG)^6	2	60	351	TGAAATGTAGTGCGTAGAACC	TCTATGCTCTGAGGACAATGAC
MacChr13-3	(AAT)^7	2	60	237	ACATTGCCTTTGCTGATTCCT	CATGAGACGAATAGACATGCCTACT
MacChr14-1	(AAT)^7	2	60	181	AGACTCTAGCCATACCAACCA	GCAACCTCTTCTTCTATCACTGA
MacChr14-2	(CTT)^6	2	60	198	AGCCTTTCCCTCCCTCTTCCT	TCCTTTGCCAACACCGTCTCTT
MacChr14-3	(AAG)^8	2	60	301	TGGCAGACACAACACGAGCAT	TGCCTCATCAGCTTCCCAAGT
MacChr14-4	(AAG)^10	2	60	276	ACCAAACACACACAGCACCAAAG	AGAACTCCGGCAAGCCATTCA
Mac001	(AT)11	2	60	412–420	GTGACTGGTGGACACCAAAAC	GCACTAGGTGTCACCCCCACTTCT
Mac002	(CT)8	2	60	283–297	CCCAACTGGGTTTGCAAGGAC	AGTAGCCGCGAGCTGATCGAAGAT
Mac003	(AT)9	2	60	258–276	TGGACCATTGAGGAGTTGGAC	TCCACCGTTTCACTTTCGTCAGCC
Mac004	(AT)11	2	60	224–240	CAAGAGTGTCCAGCGAGGGAA	GGGAGACATCATACTTTTGACACAT
Mac005	(AAG)10	2	60	331–343	CATAGCATGAGTTTCAAGGGA	ATTACAAACCCACTCTTCGATTT
Mac006	(AG)11	2	60	322–360	TTTCATCATTGATCATCATAG	GAGCTAATACTTAACCAGGTGAACA
Mac007	(CT)11	2	60	368–390	AGGCCTTGGGATGTTCCAGTG	GCAATCAACACAAGCACCTGTGGC
Mac008	(AT)10	2	60	388–398	AACGGTTATGTCAAGTGCAAC	TGACTTTAGCCCTCACTTCAAAGCC
Mac009	(AAG)13	2	60	241–244	CAACTCTCTCTCCCTCAGATT	TAAATCTATGCCACATCACTAGGC
Mac010	AG11	2	60	258-314	GCAACTGGATCAGCACATAAG	TCCGATCATAGTCTTAGCATTTCA

Mac011	(CT)9	2	60	175–199	AGAGGGCGAGATCCCTGACTC	TGAAATTTGGCGTGGGGAAAGCGT
Mac012	(AC)10	2	60	309–321	TATCAGGACCATCAACAATGA	GCCTGTTGTAGGTAAAGTGGAGAT
Mac013	(AC)6	2	60	277-317	TTTCTGAGCCAACCTATGATA	GGTTCAGCCAAAAACTTTATCAAC
Mac014	(AG)7	2	60	304-388	TAAAGGGTTCTCAATCCTTGA	GTAATTATCCCAACAGAACCCAAT
Mac016	(AAG)10	2	60	268-308	ATGGGTTCCTCGTTGGGTGGA	ACATCAAATTCGCTTGGCGTTTGCC
Mac017	(CT)8	2	60	237-271	CAAAGTAGAGGTGGACGGCCC	GGCCCTGACTGAAAGCTATGCTGG
Mac018	(TA)9	2	60	417-455	TGAGACCTTTGCTTTAGTCTG	AATTTCCCCATCATCCCCAAGGCA
Mac019	(CT)12	2	60	184-248	GCCCGTAGGAAAAGCACAGCT	AGGCTTCTTTCATTGCCAAGGCTC
MinμS0005		2	60		GCTTCAAAGACGGACGATCC	GAGGTATGTGTAATTCTCTCC
MinμS0007		2	60		CTGATTTATGATGGTAAAGGA	GGTGAATCAAAGATTAGACAAC
MinμS0008		2	60		GAACTCTAGAAGTCGAAGCAC	ACCTCTCCACCATGCATTAC
MinμS0015		2	60		TGGCTATGCTTTGATCCCTTG	GACTACTGCTTTGGGTGGTA
MinμS0016		2	60		AGCAGGTAGAGAAGGCAATC	AACCAACCCGTACAGAACCC
MinμS0037		2	60		TGTTGTAGAACGGGGTTCAC	TGTTTTCTCAGTCGCGATGG
MinμS0038		2	60		CAGCAGCAGCAACAACCACCT	AAACTCAATACCGAGGAAGC
MinμS0040		2	60		TAAGCAAGAGCACAGGGCAG	ACTCATAAGAGACCACGCCC
MinμS0048		2	60		GAGCACAAGCGGATACATTC	AGAGCACAGGTGACAATAGG

Note: Tm, melting temperature; bp, base pairs; F, forward primer; R, reverse primer.

Supplementary Table S3. The KOG annotation of 18 SSR locus.

SSR Locus	KOG Annotation	KOG Code
Mac001	Signal transduction mechanisms	*T*
Mac002	Cell cycle control, cell division, chromosome partitioning	*D*
Mac003	Lipid transport and metabolism	*I*
Mac004	Translation, ribosomal structure and biogenesis	*J*
Mac005	Posttranslational modification, protein turnover, chaperones	*O*
Mac006	Translation, ribosomal structure and biogenesis	*J*
Mac007	Signal transduction mechanisms	*T*
Mac008	Extracellular structures	*W*
Mac009	Lipid transport and metabolism	*I*
Mac010	Lipid transport and metabolism	*I*
Mac011	Posttranslational modification, protein turnover, chaperones	*O*
Mac012	Translation, ribosomal structure and biogenesis	*J*
Mac013	Signal transduction mechanisms	*T*
Mac014	Carbohydrate transport and metabolism	*G*
Mac015	Amino acid transport and metabolism	*E*
Mac016	Translation, ribosomal structure and biogenesis	*J*
Mac017	Cytoskeleton	*Z*
Mac018	Defense mechanisms	*V*

Supplementary Table S4. Allele sizes (bp) at 18 SSR loci for 104 *Macadamia* individuals.

Number	MacChr02-1	MacChr02-4	MacChr03-3	MacChr04-1	MacChr05-1	MacChr05-2	MacChr06-1	MacChr07-2	MacChr07-3	MacChr08-2	MacChr09-3	MacChr11-2	MacChr11-4	MacChr13-1	MacChr13-2	MacChr14-2	MacChr14-3	MacChr14-4
1	218	115	279	283	179	156	325	278	263	169	238	199	374	121	358	196	261	273
1	230	118	279	289	179	156	328	280	273	177	241	241	380	136	358	196	306	282
2	230	115	279	283	179	156	325	280	263	177	238	219	350	136	349	186	306	261
2	230	121	300	289	185	171	325	280	270	178	247	222	383	136	349	193	315	268
3	218	121	300	280	182	156	313	280	263	169	250	219	359	124	349	102	303	267
3	230	121	300	289	197	165	322	284	273	178	250	222	383	133	358	189	303	273
4	230	115	279	280	182	156	313	280	263	169	238	219	359	133	349	102	303	267
4	230	115	279	289	182	165	325	280	273	177	250	244	383	136	358	102	324	273
5	230	115	279	289	179	156	325	280	263	172	238	219	350	136	358	190	261	270
5	233	115	279	289	182	165	325	280	273	177	247	238	380	136	358	190	324	273
6	230	118	279	283	182	156	325	280	263	169	241	244	322	133	349	167	261	267
6	230	121	279	289	182	165	328	302	273	178	250	244	321	136	349	181	303	273
7	230	115	279	289	182	156	322	280	270	169	238	219	342	121	349	181	261	261
7	230	121	279	289	182	165	328	302	273	178	247	244	323	136	349	193	303	267
8	230	115	279	289	182	156	322	280	273	177	241	199	312	127	358	102	261	273
8	230	115	279	289	182	165	328	314	273	178	247	238	325	136	358	102	303	273
9	224	115	279	289	182	156	319	280	263	169	238	244	359	136	349	102	303	273
9	233	115	300	289	185	168	328	280	273	178	250	244	359	136	358	196	306	282
10	230	115	279	289	179	156	325	280	273	178	241	219	350	127	349	193	306	261
10	230	115	279	289	185	168	325	280	273	178	247	238	380	136	349	225	324	273
12	230	115	279	283	179	156	325	278	263	172	238	199	374	121	327	196	261	273
12	233	115	279	289	182	156	328	278	273	178	241	241	380	133	327	196	306	282
13	230	118	279	289	179	156	322	278	263	172	238	199	374	133	327	102	261	273
13	233	118	279	289	179	156	328	280	269	178	241	241	380	136	340	190	306	273
15	218	115	300	280	182	156	313	280	263	169	247	222	383	133	358	102	303	267
15	230	115	300	289	197	165	322	280	273	178	250	222	383	133	358	189	303	267
16	230	115	279	289	182	165	322	280	263	172	241	219	350	121	349	189	261	273
16	230	115	300	289	197	165	322	284	273	178	247	219	359	133	349	189	303	273
17	230	115	279	289	179	156	322	280	263	172	241	219	350	127	358	102	261	273
17	230	115	279	289	182	156	325	280	273	178	247	244	359	136	358	102	303	273
18	230	115	279	283	179	162	325	280	263	172	241	199	374	127	349	193	261	261
18	230	115	300	283	182	165	325	302	270	178	241	222	383	136	349	196	303	273
19	227	115	279	283	182	156	322	278	270	169	247	219	359	136	340	102	303	267
19	233	121	279	289	182	156	325	280	273	178	250	238	380	136	340	181	303	273
20	230	115	279	283	179	156	325	278	263	166	238	219	350	121	327	193	306	261
20	233	118	279	289	182	156	328	280	273	178	238	244	359	133	327	196	306	282
21	230	115	279	280	182	162	322	314	270	172	241	219	350	127	358	189	261	273
21	230	118	279	283	182	165	322	314	273	178	244	225	359	136	358	189	303	273

23	230	121	279	289	182	156	313	280	270	175	238	247	361	136	349	181	303	267
23	233	121	279	289	182	168	322	302	273	177	244	250	342	136	358	193	315	267
24	227	118	279	289	182	156	328	280	273	169	238	244	351	133	349	181	303	267
24	227	121	279	289	182	162	328	302	273	175	250	250	364	136	349	196	306	273
25	224	115	279	289	182	156	325	278	263	177	238	238	380	127	358	102	279	261
25	233	118	279	289	185	168	328	278	273	177	250	241	380	136	358	196	324	273
26	218	115	279	280	185	168	322	280	261	169	238	210	350	136	340	167	261	267
26	230	115	300	280	185	168	322	280	269	178	244	241	377	136	358	167	285	273
27	230	115	279	280	179	156	322	314	263	172	241	199	360	121	349	167	261	273
27	230	115	279	283	182	156	322	314	273	178	247	225	374	133	349	167	285	285
28	230	115	279	280	179	156	322	280	270	169	241	219	350	136	358	193	261	273
28	230	118	279	283	182	156	325	280	273	178	247	225	359	136	358	196	261	285
30	218	118	279	289	182	162	322	280	270	177	238	199	383	136	327	181	315	268
30	233	121	279	289	185	171	325	302	273	177	241	222	383	148	358	193	315	279
32	230	115	279	280	182	156	322	302	270	177	238	199	377	127	358	102	303	273
32	233	115	279	289	185	171	328	302	270	178	241	244	380	136	358	196	324	273
37	230	118	279	283	179	156	322	280	263	178	238	199	359	121	358	193	306	261
37	233	118	279	289	182	165	328	280	273	178	241	244	374	136	358	196	306	282
38	230	115	279	283	179	156	322	280	263	178	238	199	374	133	349	189	285	261
38	230	118	279	289	182	162	322	280	273	178	247	222	383	136	349	193	306	273
39	230	115	279	283	182	162	322	280	263	177	238	220	350	127	327	186	306	261
39	230	121	279	283	182	165	322	302	273	178	247	220	359	136	327	193	315	268
40	230	115	279	289	182	156	325	280	270	177	247	219	359	133	340	102	303	268
40	230	121	279	289	182	165	328	280	273	178	247	238	380	136	340	186	315	273
41	227	121	279	280	179	156	325	280	263	169	241	219	350	127	340	167	303	267
41	230	121	279	289	182	156	328	302	270	178	247	244	377	136	358	181	306	273
42	218	115	279	283	179	156	322	280	263	172	238	199	374	121	349	189	261	273
42	230	118	300	289	182	162	325	280	273	178	247	222	383	133	349	189	285	273
43	230	115	279	283	179	156	325	280	263	172	238	199	359	121	358	190	261	273
43	233	118	279	289	182	156	325	280	273	178	247	244	374	136	358	190	303	273
44	230	115	279	283	179	156	319	280	269	172	238	199	374	136	358	193	261	273
44	233	118	279	289	182	165	325	280	273	178	241	241	380	136	358	196	306	282
45	230	115	279	280	179	156	322	280	269	178	241	244	359	133	349	186	279	261
45	233	118	300	289	182	162	328	302	270	178	247	244	377	136	349	186	303	273
46	230	115	300	289	179	156	319	280	269	178	238	222	380	127	349	196	285	282
46	233	115	300	289	182	156	322	280	273	178	244	241	383	136	358	196	306	285
47	230	115	279	283	182	165	322	302	263	172	238	199	374	121	349	196	261	273
47	230	121	279	283	185	171	325	302	270	178	241	244	377	136	349	196	303	273
48	218	115	279	280	182	156	313	280	263	169	241	199	374	133	349	102	261	267
48	230	115	300	289	182	165	325	280	263	172	250	222	383	136	349	102	303	273
49	230	115	279	280	182	156	319	280	263	178	238	225	359	133	349	196	285	282

49	233	115	279	289	182	162	322	314	273	178	244	244	359	136	358	196	306	285
50	230	115	279	283	182	165	322	302	270	172	241	199	377	121	349	186	261	268
50	230	115	300	289	185	171	325	302	273	177	247	222	383	136	349	186	315	273
51	230	115	279	283	179	156	319	278	263	178	241	199	374	121	358	193	306	261
51	233	118	279	289	182	165	322	278	273	178	241	241	380	136	358	196	306	282
52	230	115	279	289	179	156	325	280	273	177	238	219	350	136	358	190	306	261
52	233	115	279	289	182	165	328	280	273	178	238	244	359	136	358	190	324	270
53	230	115	279	283	179	156	325	280	273	172	238	219	350	136	358	102	261	261
53	230	115	279	289	182	156	325	280	273	178	241	238	380	136	358	193	303	273
54	224	115	279	289	182	156	328	280	269	177	238	241	359	133	358	196	279	270
54	224	118	279	289	182	165	328	280	273	178	238	244	380	136	358	196	324	282
55	218	115	279	280	182	156	313	280	263	178	247	219	359	133	358	102	303	267
55	230	115	300	289	197	165	322	280	273	178	250	222	383	136	358	196	303	285
56	230	115	279	283	179	156	319	278	263	177	241	199	374	121	358	193	279	261
56	233	115	279	289	182	156	322	278	263	178	241	241	380	133	358	193	306	261
58	230	118	300	283	182	162	322	280	270	169	238	219	383	133	327	181	303	273
58	230	121	300	289	185	191	325	299	285	177	244	222	383	136	358	189	318	279
59	230	121	300	280	182	156	313	284	263	169	247	219	359	133	349	102	303	267
59	230	121	300	280	197	165	322	284	273	178	250	219	359	133	358	189	303	267
60	230	115	279	289	182	156	325	280	273	169	247	238	377	133	340	186	303	267
60	230	121	279	289	182	156	328	302	273	177	250	244	380	136	349	186	324	270
61	230	115	279	283	179	156	322	314	270	172	238	199	359	121	349	196	261	273
61	230	118	300	289	182	162	322	314	273	178	244	225	374	136	349	196	303	273
62	230	115	279	280	182	156	322	280	273	177	244	222	380	136	349	102	303	273
62	230	115	300	289	182	162	328	280	273	178	247	238	383	136	358	189	303	273
63	218	115	279	283	182	156	322	280	273	172	238	219	350	121	358	196	261	273
63	230	115	279	289	182	165	325	280	273	178	244	222	383	136	358	196	285	285
65	230	118	279	280	179	156	325	280	263	178	238	219	350	121	349	186	261	268
65	230	121	279	283	182	162	325	302	270	178	241	244	377	136	349	186	315	273
66	218	115	300	280	182	156	322	299	270	169	244	199	362	136	358	189	303	273
66	230	118	300	289	185	171	325	314	273	178	247	225	383	148	358	193	318	279
69	227	115	279	289	182	156	325	280	273	177	238	238	359	136	358	225	324	270
69	233	115	279	289	182	165	328	280	273	177	250	244	380	136	358	225	324	273
70	218	115	279	280	179	156	322	280	270	178	234	199	374	121	358	189	303	261
70	230	115	300	289	182	162	325	280	273	178	241	222	383	136	358	193	303	273
71	230	115	279	280	182	162	322	280	270	169	212	219	359	133	349	186	303	267
71	230	121	279	289	182	162	328	302	273	178	234	244	377	136	349	196	303	273
72	230	115	300	289	179	156	313	280	263	169	235	219	350	133	349	102	303	261
72	230	115	300	289	197	165	325	280	273	178	243	222	383	136	349	193	303	267
73	230	115	279	283	182	162	325	280	270	172	238	247	374	136	349	189	303	273
73	230	118	300	283	185	171	325	280	285	178	244	247	374	142	358	196	315	282

75	218	118	279	283	182	168	325	280	270	169	244	199	383	133	327	181	315	268
75	230	121	279	289	182	168	325	299	273	177	244	222	383	136	327	193	318	279
79	230	115	279	280	182	156	322	280	270	178	244	222	359	136	349	189	285	273
79	230	115	300	280	182	156	322	314	273	178	247	225	383	136	358	196	303	285
80	230	115	279	289	182	162	322	314	273	172	243	219	360	136	358	181	285	273
80	230	118	300	289	185	191	325	314	285	178	232	225	368	148	358	189	303	279
81	230	118	279	289	182	168	325	280	270	169	245	219	368	136	327	181	315	268
81	230	121	300	289	182	168	325	280	270	177	245	222	383	148	327	193	318	279
82	230	115	279	280	182	156	322	280	273	169	243	199	383	133	358	181	285	279
82	233	118	300	289	185	191	325	299	273	178	246	222	383	136	358	196	318	285
83	218	115	279	283	179	156	322	280	270	172	241	219	350	136	349	189	261	273
83	230	115	279	289	182	162	325	280	273	178	243	222	383	136	349	189	303	273
84	230	115	300	280	185	171	322	302	270	172	244	199	377	132	349	181	303	273
84	230	118	300	283	185	191	325	302	273	178	247	244	383	136	349	196	303	279
85	230	115	279	280	182	162	322	280	273	169	244	219	359	136	349	181	303	273
85	230	118	300	289	182	168	325	280	273	178	244	225	368	148	349	189	318	279
86	230	115	300	280	182	162	322	280	270	172	244	199	368	136	358	181	303	273
86	230	118	300	289	185	191	322	302	273	178	244	222	383	148	358	189	303	279
88	230	115	279	280	182	156	322	280	270	169	244	219	368	133	349	181	303	273
88	230	118	279	289	185	191	325	284	285	178	244	222	383	148	358	189	318	279
90	230	118	279	283	182	162	325	280	270	172	241	222	383	133	349	181	303	268
90	230	121	300	289	185	191	325	280	285	177	244	222	383	136	349	186	315	279
91	230	118	279	283	182	162	325	280	270	169	238	199	383	136	327	181	303	273
91	230	121	279	289	182	168	325	280	273	178	244	222	383	148	327	189	318	279
92	218	118	279	283	182	168	325	299	270	169	244	199	374	136	327	181	315	268
92	230	121	279	289	182	168	325	302	273	177	244	247	383	148	327	193	318	279
93	230	118	279	280	179	156	322	299	273	172	241	199	374	133	349	181	303	261
93	230	118	300	283	182	168	325	299	273	178	244	199	383	136	349	193	303	279
94	218	115	279	283	182	156	313	280	269	169	238	222	350	136	349	181	303	267
94	230	121	279	289	182	162	325	280	270	178	244	231	383	136	358	189	303	273
97	218	118	279	289	182	168	325	280	270	169	238	199	383	136	349	181	315	267
97	230	121	279	289	185	191	325	302	273	177	244	222	383	148	358	193	318	279
99	230	118	300	280	182	165	322	305	263	172	241	219	350	136	349	181	303	261
99	230	118	300	289	182	168	325	305	273	178	244	219	368	148	349	193	303	279
100	218	115	279	283	179	156	322	302	270	177	238	199	374	136	358	193	261	268
100	230	115	279	289	182	162	322	302	273	178	244	247	374	136	358	193	315	273
101	230	118	279	283	182	168	325	280	273	172	241	199	374	133	349	181	318	279
101	233	118	279	289	182	168	325	280	285	172	241	247	374	142	349	196	318	282
102	230	115	279	280	182	156	322	280	273	169	244	219	368	136	349	181	285	279
102	230	118	300	289	182	168	325	280	273	178	247	222	383	148	349	196	318	285
103	230	118	279	289	182	168	322	280	270	169	238	219	368	133	349	181	303	273

103	230	121	279	289	182	168	325	280	285	178	244	247	374	136	358	189	318	279
104	230	115	279	283	182	156	322	280	263	166	238	199	374	136	358	102	303	261
104	233	118	279	289	182	165	328	280	273	178	241	238	380	136	358	193	306	273
105	218	115	279	283	182	156	325	280	270	177	244	244	359	136	340	102	315	268
105	230	121	279	289	182	168	325	280	273	177	247	247	374	136	340	189	324	273
106	218	115	279	283	182	162	322	280	270	177	238	222	374	136	358	189	303	268
106	230	121	279	289	182	168	325	302	270	178	244	247	383	136	358	193	315	273
107	230	115	279	289	185	171	325	299	270	169	241	199	377	136	327	181	315	279
107	233	118	279	289	185	191	325	302	273	177	244	244	383	148	327	193	318	279
108	227	115	279	289	182	156	325	280	273	177	247	238	365	136	349	102	303	273
108	233	115	279	289	185	168	328	280	273	178	250	244	367	136	358	225	324	273
109	230	115	279	289	182	156	325	280	272	177	238	238	359	136	340	102	303	270
109	233	115	279	289	182	165	328	280	272	178	247	244	380	136	358	189	324	273
110	230	118	279	280	182	168	325	299	273	172	244	199	376	133	349	181	303	279
110	230	118	300	289	185	191	325	299	285	178	244	219	375	148	349	181	318	279
111	230	118	300	283	182	168	325	280	273	172	241	199	378	133	349	181	306	279
111	233	118	300	289	185	171	325	299	285	178	244	247	371	142	349	196	315	282
112	227	115	279	289	182	156	313	280	273	169	244	219	374	133	327	181	303	267
112	233	121	279	289	182	165	328	280	273	175	250	250	380	136	349	196	303	273
113	218	115	279	283	182	162	322	280	270	177	238	222	359	136	358	189	303	268
113	230	121	279	289	182	168	325	302	270	178	244	247	381	136	358	193	315	273
115	230	115	279	283	179	156	322	280	263	172	238	199	374	121	327	193	261	261
115	230	118	279	289	182	165	325	280	273	178	241	219	332	136	327	193	306	273
116	230	118	279	280	182	168	325	299	273	172	244	199	377	133	349	181	303	279
116	230	118	300	289	185	191	325	299	285	178	244	219	391	148	349	181	318	279
117	230	115	279	289	182	156	325	280	263	169	238	244	343	136	349	102	303	273
117	230	118	300	289	185	191	328	280	273	175	244	244	391	148	349	181	318	279
118	230	121	279	289	182	156	313	280	269	169	238	232	382	136	349	181	303	267
118	236	121	279	289	182	156	322	280	269	178	244	250	363	136	349	196	306	282
119	218	115	279	283	182	162	322	280	270	177	238	222	343	136	358	189	303	268
119	230	121	279	289	182	168	325	302	270	178	244	247	384	136	358	193	315	273
120	224	115	265	283	179	173	322	293	263	169	238	211	334	133	340	185	279	261
120	224	118	276	283	179	173	325	293	269	178	241	247	376	142	358	186	306	264
121	230	115	279	289	182	191	325	280	261	169	238	199	356	136	349	173	303	273
121	230	118	300	289	185	191	328	280	272	175	244	244	383	145	349	173	315	276
122	230	117	300	283	182	168	325	297	272	172	241	199	374	133	349	179	303	276
122	233	114	300	289	185	171	325	297	285	172	244	247	383	142	349	193	315	279
123	218	115	279	289	179	156	319	278	263	177	238	241	359	133	358	189	279	264
123	230	118	279	289	182	156	328	280	269	178	241	244	380	136	358	193	306	282
124	230	118	279	280	179	171	322	280	261	177	241	219	350	136	340	185	306	267
124	230	121	279	283	185	171	325	302	269	177	241	244	377	136	358	189	315	273

125	218	115	300	280	182	165	313	280	261	169	247	219	359	133	349	186	303	264
125	230	121	300	289	197	165	322	284	272	178	250	222	383	133	358	186	303	273
126	218	115	279	280	182	162	322	280	269	166	244	222	362	133	349	186	285	273
126	230	115	300	289	182	162	322	314	272	178	247	225	383	136	358	193	303	273
127	230	115	279	289	182	156	325	280	272	177	238	238	359	136	340	102	303	270
127	233	115	279	289	182	165	328	280	272	178	247	244	380	136	358	189	324	273

Note: Each individual is represented by two rows corresponding to its two alleles.
